# Nottingham Hip Fracture Score: Does It Predict Mortality in Distal Femoral Fracture Patients?

**DOI:** 10.7759/cureus.19139

**Published:** 2021-10-30

**Authors:** Maheswaran W Archunan, Sadhin Subhash, Joseph Attwood, Siddhant Kumar, Nameer Choudhry, James Fountain, Ignatius Liew

**Affiliations:** 1 Trauma and Orthopaedics, Norfolk and Norwich University Hospital, Norwich, GBR; 2 Trauma and Orthopaedics, Aintree University Hospital, Liverpool, GBR

**Keywords:** perioperative mortality, pre-operative management, nottingham hip fracture score, lower extremity trauma, distal femur fracture

## Abstract

Background

Patients with distal femur fractures are associated with mortality rates comparable to neck of femur fractures. Identifying high-risk patients is crucial in terms of orthogeriatric input, pre-operative medical optimisation and risk stratification for anaesthetics. The Nottingham Hip Fracture Score (NHFS) is a validated predictor of 30-day mortality in neck of femur fracture patients. In this study, we aim to investigate and evaluate the suitability of the NHFS in predicting 30-day as well as one-year mortality of patients who have sustained distal femur fractures.

Methods

Patients admitted to a level 1 major trauma centre with distal femur fractures were retrospectively reviewed between June 2012 and October 2017. NHFSs were recorded using parameters immediately pre-operatively.

Results

Ninety-one patients were included for analysis with a mean follow-up of 32 months. The mean age was 69, 56 (61%) patients were female, 10 (11%) were open fractures and 32 (35%) were peri-prosthetic fractures with 85% of patients being surgically managed. Forty-one patients were found to have an NHFS >4. Overall mortality at 30 days was 7.7% and at 1 year was 21%. Patients with an NHFS of ≤4 had a lower mortality rate at 30 days of 6% compared with those with >4 at 9.8% (p=0.422). On Kaplan-Meier plotting and log-rank test, patients with an NHFS of >4 were associated with a higher mortality rate at 1 year at 36.6% compared to patients with an NHFS of ≤4 at 8% (p=0.001).

Conclusion

NHFS is a promising tool not only in neck of femur fractures but also distal femur fractures in risk-stratifying patients for pre-operative optimisation as well as a predictor of mortality.

## Introduction

Annually, distal femur fracture accounts for 3-6% of femoral fractures, and 0.4% of all fractures [1-3]. Fragility fractures are projected to reach 21 million by 2050, which signifies a huge healthcare provision for the future [3]. The distribution of distal femur fractures is similar to proximal femur fractures, where it shares a small incidence of high-energy fracture in the younger population and is predominantly an osteoporotic low-energy fracture involving the elderly [1,2,4]. Within literature, the post-operative mortality reported ranges 7-10% at 30 days and 17-30% at one year [1,2,4,5]. Furthermore, neck of femur fracture and distal femur fracture patients share similar mortality rates [2,4].

The National Hip Fracture Database was established with the recent introduction of Best Practice Tariff and played a huge role in improving care for neck of femur fracture patients [2,5-7]. We have observed an improvement in mortality rates from 10.9% in 2007 to 6.7% in 2017 [6]. Besides that, a multidisciplinary team (MDT) approach to optimising this group of patients with pre-existing morbidities has better stratified high-risk patients for optimisation to surgery [8]. The Nottingham Hip Fracture Score (NHFS) is utilized as a scoring system in predicting postoperative mortality at 30 days and is yet to be described for other cohort of patients [8]. Currently, there are no standardised scores to risk-stratify patients with distal femur fractures, with aid to improve outcome in a standardised national guideline [1].

We aim to investigate the suitability of the NHFS in predicting the risk of 30-day and one-year mortality in patients who have sustained distal femur fractures.

This work was previously presented as an abstract (https://www.britishtrauma.com/wp-content/uploads/2021/04/Archer-Yates-BTS-Programme-2018-FINAL.pdf; https://58society.com/2018/06/25/nottingham-hip-fracture-score-predict-mortality-distal-femoral-fracture-patients/; and https://www.boa.ac.uk/asset/83BBE23F-5F58-4872-927033FF82F0F485/).

## Materials and methods

From June 2012 and October 2017, all patients admitted to a level 1 major trauma centre with distal femur fracture were included and retrospectively reviewed, including patients who sustained peri-prosthetic fractures and those who were conservatively managed. The 30-day and one-year postoperative mortalities were recorded from the electronic patient record system MedWay. MedWay is a reliable system at reporting any deaths as it is synchronised with data from the community and is therefore updated regularly. 

Patient demographic data and NHFS were recorded using parameters immediately pre-operatively. The parameters required to calculate the NHFS include age, gender, Abbreviated Mental Test Score (AMTS), haemoglobin on admission, residence, number of co-morbidities and history of active malignancy in the last 20 years (Table 1). Patients are given a score accordingly, with a maximum score being 10. The score is subsequently used to predict the risk of 30-day mortality (Table 2). 

**Table 1:** Nottingham Hip Fracture Score - patient parameters Hb, haemoglobin; AMTS, Abbreviated Mental Test Score.

Variable	Value	Score
Age	<66	0
	66-85	3
	>86	4
Gender	Male	1
Admission Hb	≤100 g/L	1
AMTS	≤6	1
Living in an institution	Yes	1
Number of comorbidities	>2	1
Malignancy	Yes	1

**Table 2:** Predicted 30-day mortality according to total score

Total Nottingham Hip Fracture Score	Predicted 30-Day Mortality (%)
0	0.7
1	1.1
2	1.7
3	2.7
4	4.4
5	6.9
6	11
7	16
8	24
9	34
10	45

Accuracy of data was sampled and verified by the senior authors with an error rate of <5%. Patients were stratified into high and low risk with an NHFS of >4 and ≤4, respectively (Table 3). This was previously shown in the literature with divergence of survival curve in survival rates of neck of femur fracture patients [8]. Besides that, patients above the age of 65 who were selected for conservative management due to high anaesthetic risk were compared to those surgically managed to examine their survival. 

**Table 3:** Patient stratification *p-Value <0.05. NHFS, Nottingham Hip Fracture Score.

	Total (n=91)	NHFS ≤4 (n=50)	NHFS >4 (n=41)	p-Value
Age (years)	69 (16-101)	57±23 (16-101)	84±8 (64-95)	<0.0001*
Age ≥65	30/91 (33%)	30 (60%)	40 (98%)	0.0001*
Surgical fixation	78/91 (87%)	46 (92%)	32 (78%)	0.075
Mortality rate at 30 days	7 (7.7%)	3 (6%)	4 (9.8%)	0.422
Mortality rate at 1 year	19 (21%)	4 (8%)	15 (36.6%)	0.001*

Statistical analysis 

Data were collected using electronic patient record system MedWay and radiographs reviewed on Kodak Carestream PACS system (Kodak, UK). Data analysis was performed using GraphPad Prism 7. Shapiro-Wilk test was used to determine normality with parametric data analysed using student's t-test and Fisher’s exact test. We utilized the log-rank test to compare the Kaplan-Meier curves. Statistical significance was determined with a p-value of 0.05.

## Results

Ninety-one patients were included for analysis with a mean follow-up of 32 months. The mean patient age was found to be 69 (range 16-101). Fifty-six (61%) patients were female. Ten (11%) were open fractures and 32 (35%) were peri-prosthetic fractures. Out of those included in the study, 78 patients were treated with surgical fixation (87%). 

**Figure 1:**
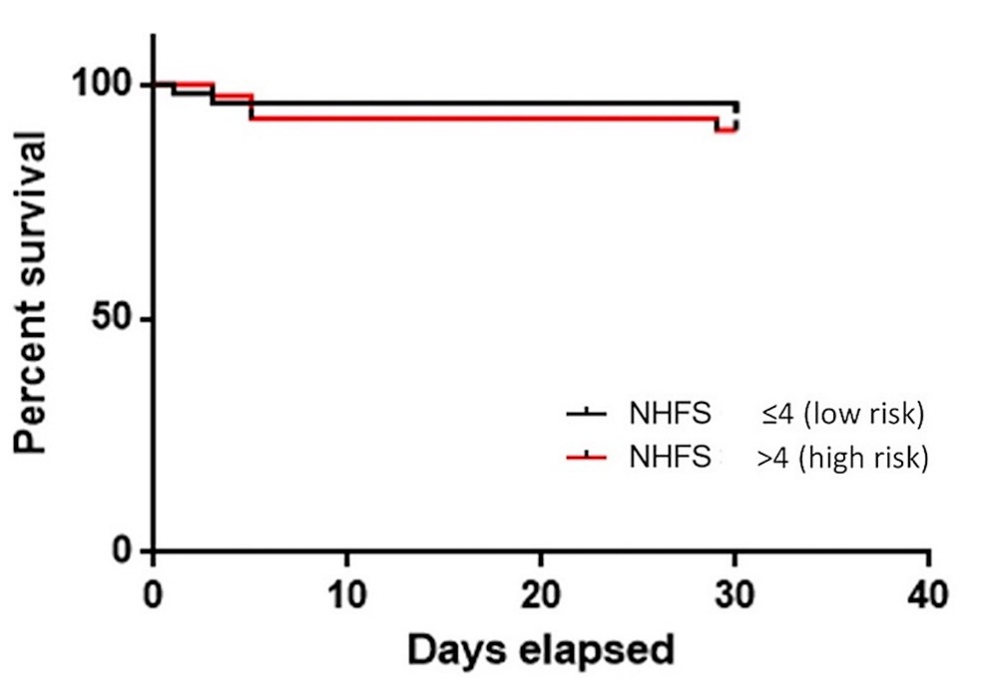
The Kaplan-Meier curve showing 30-day postoperative mortality in patients who have sustained a distal femur fracture. NHFS, Nottingham Hip Fracture Score.

**Figure 2:**
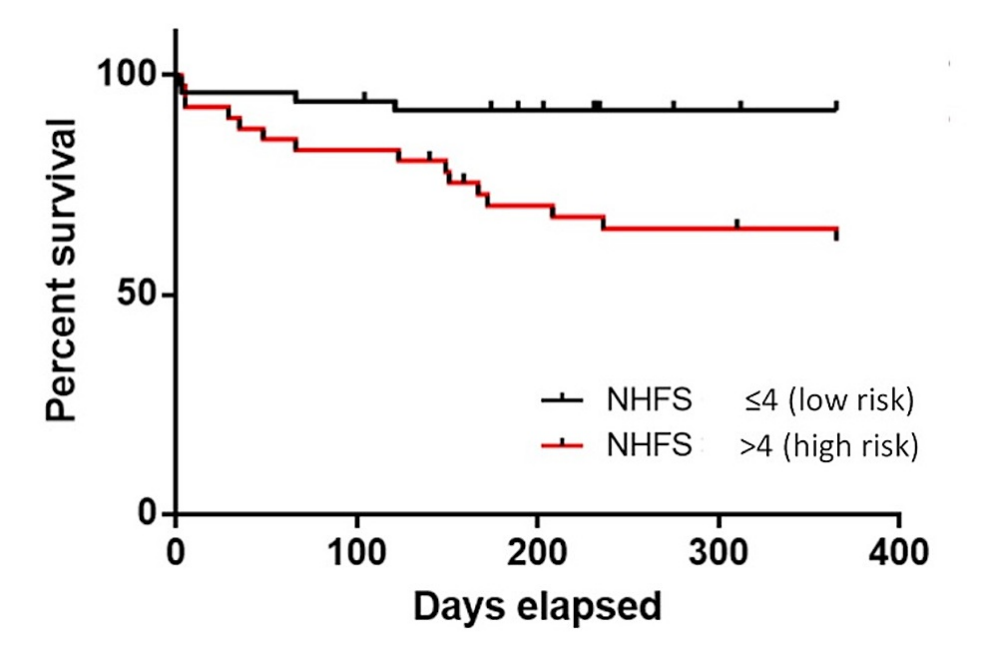
The Kaplan-Meier curve showing one-year postoperative mortality in patients who have sustained a distal femur fracture. NHFS, Nottingham Hip Fracture Score.

Within our patient cohort, the overall mortality at 30 days was 7.7% and at one year was 21%. Kaplan-Meier curves were plotted for 30-day and one-year mortality (Figures 1, 2). Forty-one patients were found to have an NHFS of >4 (45%). Patients with an NHFS of ≤4 had a lower mortality rate at 30 days with risk being 6% compared with those with >4 at 9.8% (p=0.422). On Kaplan-Meier plotting, patients with an NHFS of >4 were associated with a higher mortality rate at one year at 36.6% compared to patients with an NHFS of ≤4 at 8% (p=0.001) as illustrated in Figure 2.

 

**Figure 3:**
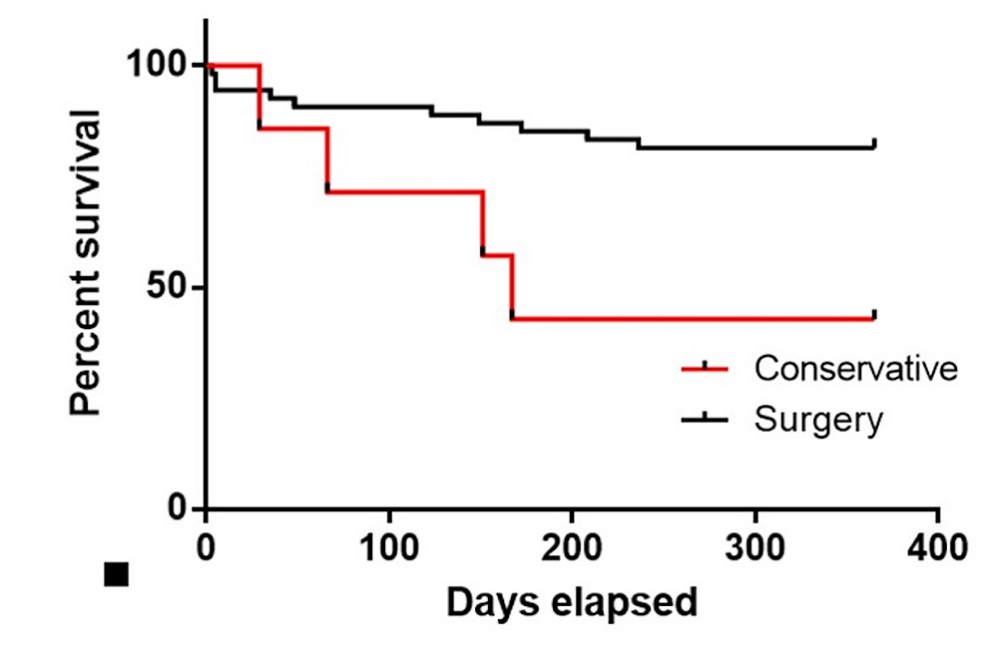
The Kaplan-Meier curve showing postoperative mortality between two groups: conservatively managed patients versus surgically managed patients in age ≥65 years.

Patients above the age of 65 who were conservatively managed were compared with those receiving surgical fixation, which showed a mortality rate of 14% and 4%, respectively, at 30 days and 57% and 18.5% at one year as illustrated in Figure 3. All patients who were conservatively managed had an NHFS of >4 suggesting that they were at high risk to undergo any surgical intervention. 

## Discussion

This study has demonstrated that the NHFS can be used to identify and stratify high-risk patients who have sustained a distal femur fracture. Patients with an NHFS of >4 had a 28% greater mortality rate at one year. Without utilising a risk score, patients above the age of 65 years who were reviewed as at high anaesthetic risk were managed conservatively (with hinged knee brace), in which all had an NHFS of >4 retrospectively.

Being able to stratify these high-risk patients provides a stratification tool to improve delivery of care from the MDT for distal femur fractures in terms of orthogeriatric input, pre-operative anaesthetic assessment and post-operative critical care optimisation which is often neglected [1]. With risk stratifications, it also acts as an audit tool for national databases, communication with colleagues and patients regarding outcomes, as well as for further research [1,5,8,9]. NHFS can also be used as a comparative tool amongst different units for mortality outcomes [6,8]. Besides that, in our experience, NHFS can be used as an adjunct to an MDT approach, as it is able to identify high-risk patients, especially those deemed as at high anaesthetic risk with fracture configurations amenable to conservative management. 

NHFS is simple to utilise when compared to other scores that were originally not validated for orthopaedic patients such as P-POSSUM, Charlson Comorbidity Index and Estimation of Physiologic Ability and Surgical Stress (E-PASS) [10,11]. The NHFS is suggested as the score showing the most promising results [11]. More importantly, the NHFS has been validated and modified following multiple studies, namely involving three UK hip fracture units: Peterborough (1992-2009), Brighton (2008-9) and Nottingham (2000-9) [12]. However, further research is on-going in examining the validity of other scores when applied specifically to distal femur fractures.

Limitations of this study include the small cohort for analysis, which will require further validation with multicentre, international cohort of patients. The entirety of retrospective data collection and accuracy of data relies on the accuracy of medical records on admission, which is improved by our electronic patient record system as well as cross-checking by senior authors. As our institution is the major trauma centre for the region, some patients (especially the younger cohort) may have sustained concomitant injuries, which may have affected their outcome. However, our mortality rates were found to be similar to those described within the literature and there were no early mortality due to major trauma [1,2,4].

## Conclusions

The NHFS was originally developed and introduced to predict 30-day mortality in patients who have sustained a neck of femur fracture. The score has been validated through several subsequent studies and has been found to be a reasonably accurate predictor of 30-day mortality. In this study, we addressed whether the NHFS can be utilised to risk-stratify patients who have sustained a distal femur fracture as they are also at risk of developing multiple complications leading to death. From our study, we have found that the NHFS is a promising tool to risk-stratify distal femur fracture patients and identifying those with high risk of mortality. 
